# Bidirectional regulation of the ubiquitin-RNA modification axis in cancer

**DOI:** 10.3389/fonc.2026.1761698

**Published:** 2026-04-10

**Authors:** Kailang Li, Lingling Bao, Bitao Jiang, Xiaofeng Jin

**Affiliations:** 1 Department of Oncology, Beilun Branch of the First Affiliated Hospital, College of Medicine, Zhejiang University, Ningbo, China; 2 Department of Oncology, Beilun People’s Hospital, Ningbo, China; 3 Department of Biochemistry and Molecular Biology, Health Science center, Ningbo University, Ningbo, China

**Keywords:** cancer, epitranscriptomics, network, RNA modifications, ubiquitination

## Abstract

RNA modifications, dynamically regulated by RNA-modifying proteins (RMPs) acting as “writers”, “erasers”, and “readers”, play pivotal roles in governing gene expression and cellular fate. These modifications are also intimately linked to cancer initiation and progression. Dysregulation of RMPs in tumors disrupts RNA modification homeostasis, thereby promoting cancer progression through enhanced proliferation, metastasis, and immune evasion. The ubiquitination system serves as critical regulator of RMP stability and activity, which in turn shapes the cancer epitranscriptome. Conversely, RNA modifications feedback into ubiquitination pathways by modulating the stability and translation of mRNAs encoding ubiquitination-related factors. This bidirectional crosstalk between RMPs and ubiquitination forms a sophisticated regulatory network that enhances cancer adaptability. Notably, emerging therapeutic strategies aimed at targeting RMP ubiquitination have shown promising potential. In this review, we systematically examine the bidirectional regulatory axis between ubiquitination and RMPs in cancer pathogenesis. We first outline how ubiquitination controls RMP activity and the consequent epitranscriptomic alterations and then explore how RNA modifications reciprocally influence ubiquitination pathways. Building on this mechanistic foundation, we evaluate current therapeutic approaches targeting the ubiquitination-epitranscriptome axis and highlight key knowledge gaps in our understanding of this dynamic regulatory network. Finally, we propose future research directions to fully decode the therapeutic potential of this dynamic regulatory network in oncology, thereby providing novel perspectives on cancer development.

## 1 Introduction

The study of cancer has evolved through distinct paradigms from early genomic explorations of oncogenic mutations to the epigenetics era focusing on DNA methylation and histone modifications (1). While these advances have identified critical drivers of tumorigenesis, they cannot fully account for the profound heterogeneity and plasticity observed in cancers. The emergence of epitranscriptomics about the study of RNA chemical modifications has revealed a crucial new layer of regulatory complexity in cancer biology (2). Over 170 RNA modifications have been identified which dynamically fine-tune RNA metabolism to influence cellular processes ranging from stress adaptation to malignant transformation (3).

**Figure 1:**
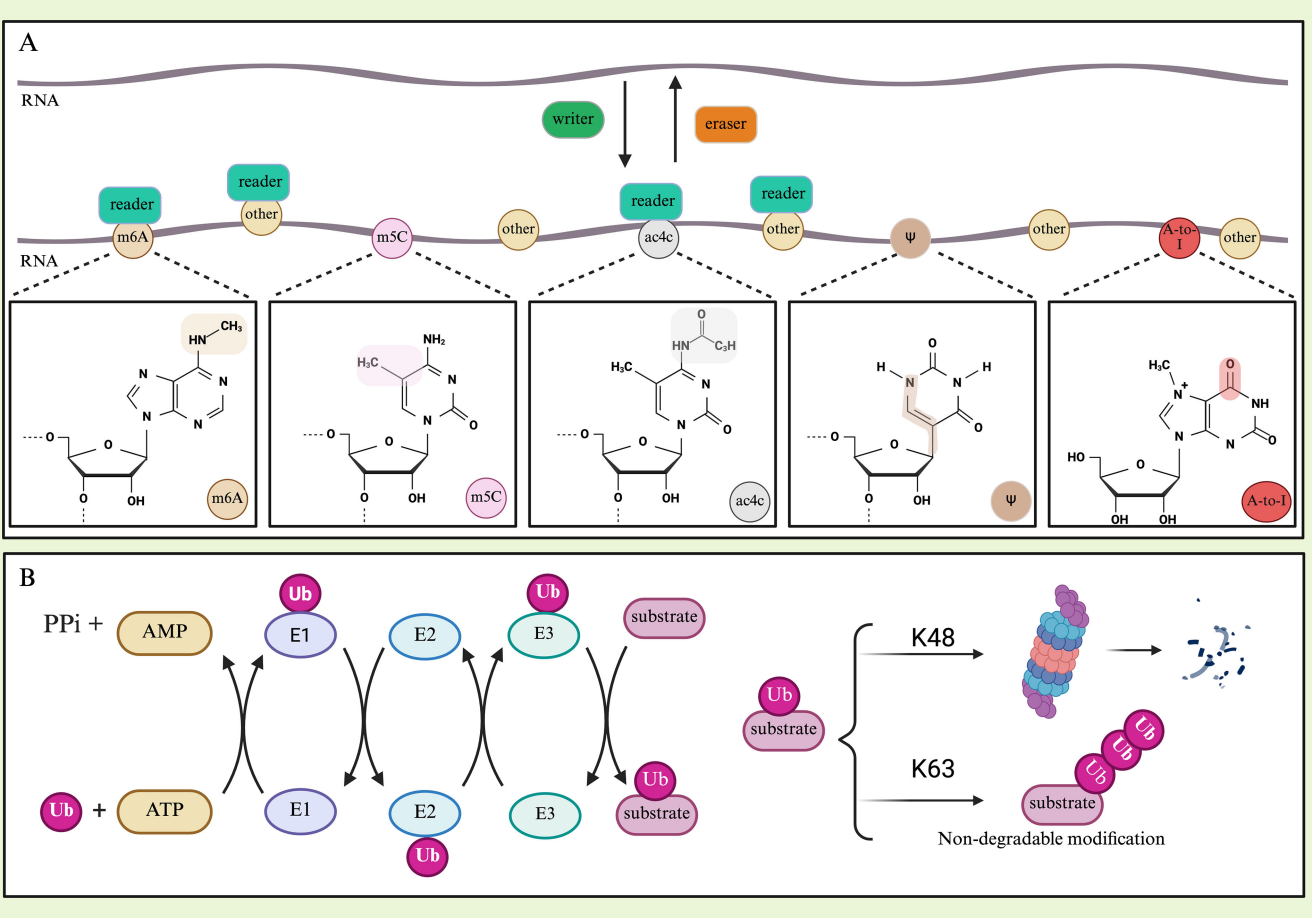
**(A)** RNA modification is controlled by RNA modifying proteins (RMPs): writers install modifications, erasers eliminate them, and readers interpret their functional consequences. Simultaneously displaying the chemical structures of the four RNA modifications mentioned in this article. m6A N6-methyladenosine, m5C 5-methylcytosine, ac4C N4-acetylcytidine, ψ pseudouridine, A-to-I edited adenosine to inosine. **(B)** Ubiquitination process. First, ubiquitin E1 enzyme activates by ATP and binds to Ub. Then, the Ub-E1 intermediate transfers this activated Ub to the ubiquitin E2 enzyme, after which it is transferred Ub to the specific substrates via E3 ubiquitin ligases. E3 ligases and transfer Ub to substrates. Ubiquitination may lead to degradation of substrate protein, or nondegradable modification. Due to the presence of seven lysine residues (Lys6, Lys11, Lys27, Lys29, Lys33, Lys48, Lys63) and one N-terminal methionine (M1) on the Ub molecule, eight different types of ubiquitin chains can be formed. The most common types are K48 and K63. K48 ubiquitination modification often leads to degradation of substrate proteins, while K63 ubiquitination modification causes functional changes in substrate proteins.

RNA modifications act as dynamic switches that control RNA fate and function. At the most fundamental level, 5’-cap modification and the poly(A) tail are common structures found in eukaryotic mRNA, controlling stability and facilitating translation. Beyond these terminal modifications, internal RNA modifications, including N6-methyladenosine (m6A), 5-methylcytosine (m5C), N4-acetylcytosine (ac4C), Adenosine-to-inosine editing (A-to-I RNA editing) and pseudouridine (Ψ), have emerged as critical regulators of gene expression (**Figure 1**) (1, 3, 4). The most prevalent and well-studied internal modification is m6A, which affects RNA processing, structure, splicing, localization, stability, degradation, and translation, while also modulating RNA-protein and RNA-RNA interactions (5, 6). M5C, present in both mRNA and non-coding RNAs, plays similar roles to m6A in regulating RNA stability, translation, and nuclear export (4). Ac4C, the only known RNA acetylation in eukaryotes, promotes mRNA stability and translation efficiency (7). A-to-I editing, catalyzed by ADAR enzymes, recodes genetic information leading to protein diversity and affects RNA structure and stability (3, 8). Ψ, known as the “fifth nucleotide,” is abundant in non-coding RNAs such as tRNAs and rRNAs where it stabilizes RNA structure, and has also been found in mRNA (9).

These RNA modifications are dynamically and reversibly regulated by a tripartite system of proteins or complexes, with m6A serving as the best-characterized paradigm (10). The “writers” are responsible for installing modification molecules, while the “erasers” remove them—these are typically pairs of enzymes with opposing functions, such as methyltransferases and demethylases (3). The modification sites can then be recognized and bound by “readers,” which interpret the functional consequences and determine RNA fate (3).

For m6A specifically, the writer complex is multi-subunit. METTL3 (Methyltransferase-like 3) possesses enzymatic activity, while its allosteric homolog METTL14 exhibits higher methyltransferase activity and is critical for target RNA recognition and binding (11). WTAP (Wilms’ tumor 1-associating protein), lacking catalytic domains, interacts with METTL3 and METTL14 to affect their activity and accumulation in nuclear speckles (12). Additional m6A writers in mammals include METTL16, RBM15 (RNA binding motif protein 15) and ZCCHC4 (1). These methyltransferases exhibit sequence specificity—RRACH (R=G/A; H=A/C/U) represents a consensus methylated site with high conservation (4, 13). The m6A erasers are demethylases: FTO (fat mass and obesity-associated protein) and ALKBH5 (5, 6). They reverse m6A modification, adding another layer of dynamic control. The m6A readers recognize and bind m6A sites to mediate functional outcomes. Identified readers include YTH domain proteins (YTHDF1-3/YTHDC1-2), HNRNPs (Heterogeneous nuclear ribonucleoproteins) family, IGF2BPs (Insulin-like growth factor 2 mRNA-binding proteins) (5, 6).

While less extensively characterized than m6A, writers, erasers, and readers for other modifications have been identified. For m5C, the primary writer is NSUN2 (NOP2/Sun domain 2), with additional family members such as NSUN6, while the reader YBX1 (Y-box binding protein 1) recognizes m5C-modified mRNAs to regulate their stability (4). For ac4C, NAT10 (N-acetyltransferase 10) is the sole known writer (7). For A-to-I editing, ADAR1 and ADAR2 catalyze adenosine deamination (3, 8). For Ψ, DKC1 (dyskerin) functions as the primary synthase, often guided by snoRNAs (9).

Critically, all RMPs—whether writers, erasers, or readers—are themselves proteins whose activity, stability, and interactions are stringently controlled by ubiquitination. Ubiquitination is carried out by a cascade of enzymes: E1 activating, E2 conjugating, and E3 ligating (**Figure 1**) (14, 15). Notably, E3 ligases are key determinants of substrate specificity. Ubiquitin chains of different linkages confer distinct functional outcomes: K48-linked polyubiquitination serves as the canonical degradation signal, targeting substrates to the 26S proteasome for proteolysis, thereby precisely controlling protein stability and abundance; K63-linked polyubiquitination functions primarily as a non-degradative signal, modulating target protein activity, altering subcellular localization, or serving as a scaffold for signaling complex assembly (16). Notably, E3 ligases are often dysregulated in cancer (17). For example, TRIM21 (Tripartite motif-containing 21) acts as an E3 ligase that targets METTL3 and IGF2BP2 for proteasomal degradation, thereby suppressing their tumorigenic effects (18, 19). These findings position ubiquitination as a master regulator of epitranscriptomic machinery.

Recent studies have highlighted the reciprocal relationship between RNA modifications and ubiquitination, revealing that E3 ligase mRNAs themselves are subject to RNA modifications. For example, m6A modification promotes the degradation of E3 ligase *TRIM21* mRNA, followed by ubiquitination and degradation of RMPs by TRIM21 to reshape the transcriptome (18–20). This feedback mechanism directly couples E3 ligase dysfunction to the stabilization of oncogenic RMPs, thereby driving tumor progression. The bidirectional crosstalk forms a sophisticated regulatory network that cancer cells hijack to enhance adaptability, drive progression, and evade therapy.

In this review, we delineate the ubiquitination-RMP regulatory network and its crosstalk with RNA modifications in cancer. We first explore how ubiquitination controls RMP activity to shape the epitranscriptome. We then examine how RNA modifications reciprocally regulate ubiquitination pathways through epitranscriptomic control of E3 ligase mRNAs. Finally, we evaluate current therapeutic approaches targeting this axis and highlight key knowledge gaps and future directions.

## 2 The network for ubiquitination and RNA modification in cancer

### 2.1 The ubiquitination of RMPs in m6A

**Figure 2:**
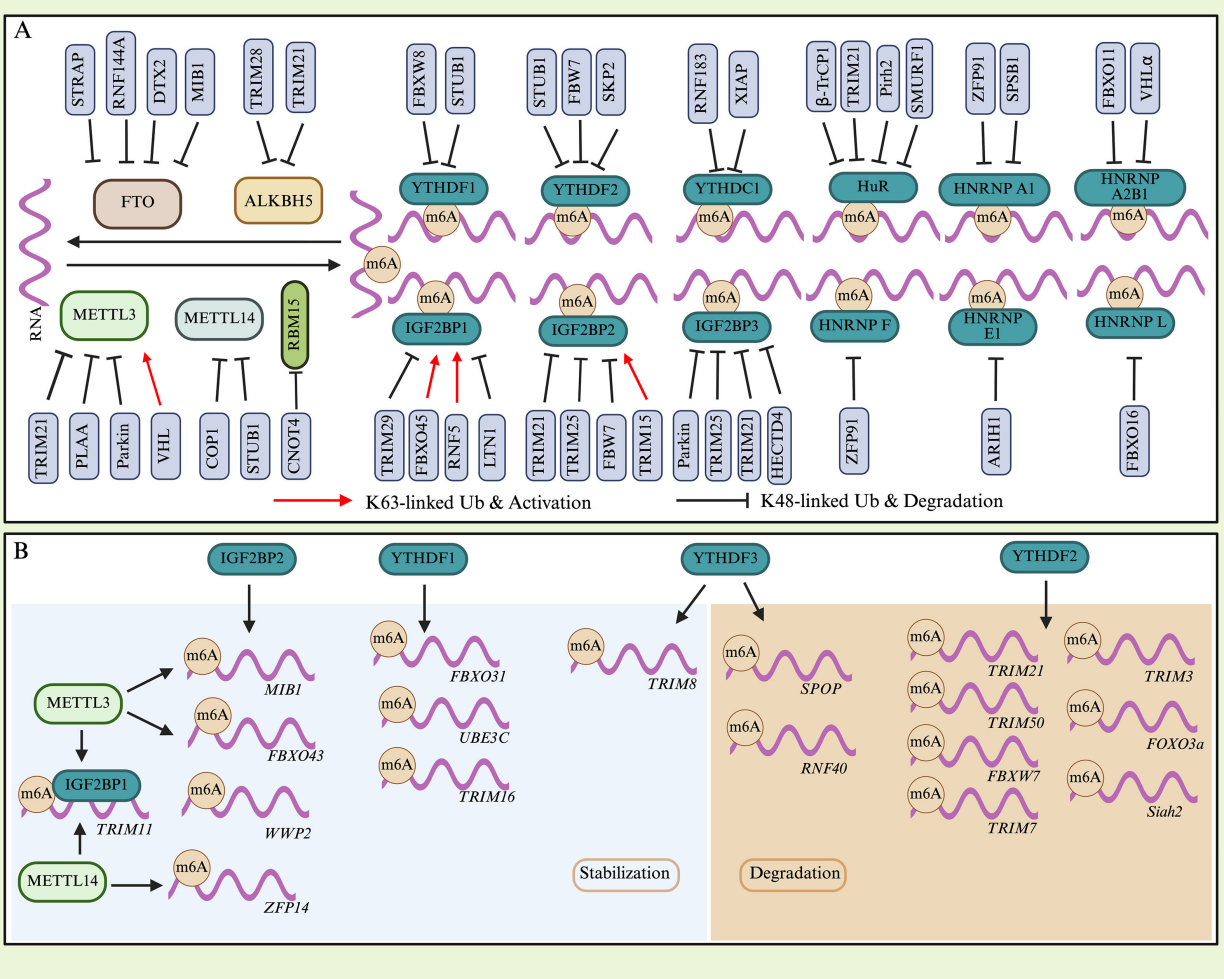
**(A)** Ubiquitination precisely controls the stability and activity of core RMPs through targeted degradation or functional remodeling, thereby globally modulating m6A modification landscape and driving tumorigenesis. **(B)** RMPs regulate the ubiquitin system by controlling the stability and translation of E3 ligase mRNA.

Ubiquitination-mediated regulation of m6A-related RMPs plays a critical role in cancer pathogenesis. Through targeted degradation or functional remodeling, ubiquitination precisely controls the stability and activity of core m6A regulators, thereby globally modulating the m6A modification landscape and driving tumorigenesis (**Figure 2** and **Table 1**).

**Table 1:** Ubiquitin-mediated regulation of m6A RMPs in cancer.

RMP category	Target protein	E3 ligase/regulator	Ubiquitination site	Cancer type	Effect on RMP	Functional outcome	Refs
Writer	METTL3	TRIM21	K459	PC	Degradation	Promote ubiquitination degradation of METTL3, thereby downregulating the stability of *SLC7A11* mRNA and inhibiting the progression of PC	(18)
		VHL	–	RCC	Stabilization	Promote the formation of METTL3/METTL14 complex through interaction with METTL3/14	(24)
		PLAA	–	OC	Degradation	Promote ubiquitination degradation of METTL3, thereby downregulating the stability of *TRPC3* mRNA and inhibiting the progression of OC	(29)
		Parkin	–	EBV related tumors	Degradation	Resulting in reduced m6A modification of *TLR9* mRNA, inhibiting anti-tumor immunity, and promoting viral carcinogenesis.	(30)
	METTL14	COP1-TRIB2 axis	–	ESCC	Degradation	Promote tumor progression	(23)
		STUB1	K148, K156, K162	CCA	Degradation	Inhibit tumor progression	(25)
	RBM15	CNOT4	–	acute megakaryoblastic leukemia	Degradation	Promote degradation of RBM15, thereby regulating selective RNA splicing and promoting tumor progression	(28)
Eraser	FTO	STRAP	K216	CRC	Degradation	Resulting in enhanced m6A modification of *MTA1* mRNA, followed by stabilization of its mRNA and promoting CRC metastasis.	(31)
		RNF144A	–	ESCC	Degradation	Suppress tumor development	(33)
		DTX2	K162	–	Degradation	Inhibits tumorigenesis and enhances anti-tumor immunity by stabilizing *LIF* mRNA	(34)
		Induced by DNA damage		PCa	Degradation	Promote the growth of PCa	(35)
		MIB1	K216	Malignant glioma	Degradation	Stabilize *CSF3* mRNA, promotes neutrophil infiltration and NETosis, ultimately leading to resistance to oHSV therapy	(36)
	ALKBH5	TRIM28 (via PRMT5 methylation)	–	CRC	Degradation	Downregulation of ALKBH5 leads to increased m6A modification of *CD276* mRNA to activate CD276 transcription, thereby increasing CRC immune escape.	(32)
		TRIM21	–	BCa	Degradation	Inhibit the progression of BCa, promote the decay of ESPL1 mRNA by degrading ALKBH5	(37)
Reader	YTHDF1	FBXW8	K469, K473, K500, K527	HCC	Degradation	Increase the expression of PD-L1 and inhibiting the expression of immune related genes, thereby promoting immune evasion in HCC cells.	(39)
		STUB1	–	RCC	Degradation	Promote the migration and invasion of RCC cells	(40)
	YTHDF2	STUB1	K245	HCC	Degradation	Inhibition of HCC proliferation and sorafenib resistance	(41)
		FBW7	–	OC	Degradation	Inhibit anti-cancer function	(42)
		SKP2	–	pMMR CRC	Degradation	Reduce the degradation of *CXCL10* mRNA enhances the infiltration of cytotoxic T lymphocytes into tumors.	(43)
	YTHDC1	RNF183	–	BC	Degradation	Reduce the stability of *JUND* mRNA, thereby inhibiting the sensitivity of bladder cancer cells to Enfortumab vedotin	(45, 46)
		XIAP	K372, K469, K472	BC	Degradation	Promote the metastasis of BC cells	(44)
	IGF2BP1	TRIM29	K440, K450	GC	Degradation	Reduce the stability of *PD-L1* mRNA, thereby enhancing the anti-tumor T cell immunotherapy effect in GC.	(48)
		FBXO45	K190, K450	HCC	Stabilization	Promote K63 ubiquitination of IGF2BP1, leading to upregulation of *PLK1* expression and HCC cell proliferation.	(51)
		RNF5	K190, K450, K465	Fatty HCC	Stabilization	Promote K63 ubiquitination of IGF2BP1, thereby enhancing the stability of *CPT1A* mRNA and promoting the progression of fatty HCC.	(50)
		LTN1	–	HCC	Degradation	Inhibition of HCC cell growth	(49)
	IGF2BP2	TRIM21	–	CRC	Degradation	Inhibition of CRC cell growth	(19)
		TRIM25	–	PC	Degradation	Promote IGF2BP2 degradation, downregulate *MEKK1* mRNA stability, inhibit PC cell proliferation and invasion	(57)
		FBW7	–	LC	Degradation	Promote the ubiquitination degradation of IGF2BP2, thereby downregulating the stability of *SLC7A5* mRNA and inhibiting radiation resistance in LC.	(53)
		TRIM15	K462, K487	PC	Stabilization (Phase Separation)	Promote K63 ubiquitination of IGF2BP2 and enhances its RNA binding activity, thus stabilizing *TLR4* mRNA, thus promoting the progress of PC and chemoresistance.	(52)
	IGF2BP3	Parkin	–	CC	Degradation	Inhibit tumorigenesis	(54)
		TRIM25	–	GC	Degradation	Promote the ubiquitination degradation of IGF2BP3, thereby inhibiting the stability of *CCND1* mRNA and inhibiting GC proliferation	(55)
		TRIM21		CRC	Degradation	Promote the ubiquitination degradation of IGF2BP3, with the assistance of BBR, thereby inhibiting the stability of *CDK4/CCND1* mRNA and promoting G1/S phase arrest in CRC cells.	(137)
		HECTD4	–	Gliomas	Degradation	Inhibit tumorigenesis	(56)
	HuR	β-TrCP1	–	Multiple Tumors	Degradation	Inhibit tumorigenesis	(63–65)
		TRIM21	K182	Glioblastoma	Degradation	Promote HuR degradation, thereby inhibiting the NF-κB and STAT3 signaling pathways to suppress the progression of glioblastoma	(68)
		Pirh2	–	LC	Degradation	Inhibit the progression	(69)
		SMURF1	–	Pituitary Tumors	Degradation	Inhibit tumorigenesis	(71)
	HNRNP F	ZFP91	K185	GC	Degradation	Inhibit the invasion and metastasis of GC	(73)
	HNRNP A1	ZFP91	K8	HCC	Degradation	Degrade hnRNP A1, altering the alternative splicing of *PKM* mRNA, thereby inhibiting aerobic glycolysis, proliferation, and metastasis of HCC cells.	(74)
		SPSB1	K183, K298	Cervical cancer	Non-degradation	Catalyze K29 ubiquitination of hnRNP A1, inducing its nuclear export and promoting alternative splicing of target mRNAs to accelerate tumor progression	(75)
	HNRNP A2B1	FBXO11	K120, K137, K168, K173	HCC	Degradation	Degrade hnRNP A2B1, regulate lipid metabolism reprogramming, and inhibit HCC progression	(76)
		VHLα	K274, K305	RCC	Degradation	Inhibit tumorigenesis	(77)
	HNRNP E1	ARIH1	K314	BCa	Degradation	Inhibit tumorigenesis	(78)
	HNRNP L	FBXO16	–	OC	Degradation	Inhibit tumorigenesis	(79)

#### 2.1.1 The ubiquitination of writers in m6A

The METTL3/METTL14 complex functions as the core m6A writer through synergistic interactions where METTL3 directly binds S-adenosylmethionine (SAM) to catalyze methylation while METTL14 stabilizes RNA substrate recognition and catalytic conformation (21). Both subunits are frequently overexpressed in cancers and function as oncoproteins via m6A-dependent manner (22). Their stability exhibits cancer type-specific regulation: TRIM21 mediates K48-linked ubiquitination of METTL3 at K459 in pancreatic cancer (PC) (18), triggering proteasomal degradation and ferroptosis induction, whereas the COP1 (Constitutive photomorphogenic 1)-TRIB2 (Tribbles homolog 2) axis drives METTL14 degradation in esophageal squamous cell carcinoma (ESCC) (23). Conversely, VHL (Von Hippel-Lindau) enhances METTL3/14 complex assembly via its E3 ligase domain in renal cell carcinoma (RCC) (24). In cholangiocarcinoma (CCA) cell, STUB1 (STIP1 homology and U-box containing protein 1) competes with METTL3 for METTL14 binding, promoting ubiquitination at multiple sites including K148/K156/K162 (25).

The scaffold protein WTAP which mediates the assembly of the m6A writer complex and recruits factors like RBM15, is subject to dynamic post-translational control. Its ubiquitination status is modulated by key tumor microenvironmental cues: promoted under hypoxic conditions but suppressed under high-glucose conditions (26, 27), thereby influencing its function in cancer progression. Notably, in acute megakaryoblastic leukemia, PRMT1 (Protein Arginine Methyltransferase 1)-mediated arginine methylation of RBM15 at R578 primes CNOT4 (CCR4-NOT transcription complex subunit 4)-dependent ubiquitination, exemplifying cross-regulation between RNA modification and ubiquitination pathways (28).

Further extending this complexity, the PLAA (phospholipase A-2-activating protein) is typically downregulated in ovarian cancer (OC), where its reduced expression inhibits ubiquitin-mediated degradation of METTL3 (29). This stabilization leads to increased METTL3 protein abundance, enhanced m6A modification of *TRPC3* (*Transient receptor potential channel subfamily members 3*) mRNA, and ultimately facilitates OC cell metastasis (29). Conversely, in Epstein-Barr virus (EBV)-associated malignancies including nasopharyngeal carcinoma (NPC), gastric cancer (GC), and B-cell lymphoma, the viral oncoprotein EBNA1 (Epstein-Barr nuclear antigen 1) promotes Parkin-mediated ubiquitination and degradation of METTL3 (30). This suppression of m6A modification reduces m6A methylation of *TLR9 (Toll-like receptor 9)* mRNA, resulting in diminished anti-tumor immunity and facilitating viral oncogenesis (30).

#### 2.1.2 The ubiquitination of erasers in m6A

As core erasers, FTO and ALKBH5 catalyze m6A demethylation to dynamically regulate RNA modifications, with their functions stringently constrained by ubiquitination in a cancer type-specific manner. In colorectal cancer (CRC), STRAP (Serine-threonine kinase receptor-associated protein) mediates K48-linked ubiquitination of FTO at K216, reducing its tumor-suppressive function (31), while PRMT5 methylation of ALKBH5 at R316 triggers TRIM28-mediated degradation, synergistically promoting tumor progression (32). Similarly, RNF144A (Ring finger protein 144A)-mediated FTO degradation suppresses tumor development in ESCC (33). DTX2 (Deltex 2) inhibits tumorigenesis and enhances anti-tumor immunity by promoting the ubiquitination and degradation of FTO (34). Notably, DNA damage-induced FTO ubiquitination in prostate cancer (PCa) enhances PARP inhibitor (PARPi) sensitivity, revealing therapeutic synergism (35). In gliomas, MIB1 (Mindbomb E3 ubiquitin protein ligase 1) induces ubiquitin-mediated degradation of FTO at K216, resulting in m6A hypermethylation-dependent CSF3 overexpression and subsequent neutrophil extracellular trap (NET) formation, thereby compromising oncolytic virotherapy efficacy (36). Conversely, TRIM21-mediated ALKBH5 degradation drives breast cancer (BCa) progression (37), highlighting the context-dependent regulatory logic of erasers.

#### 2.1.3 The ubiquitination of readers in m6A

The m6A reader protein converts epigenetic transcriptome information into differential biological outputs by recognizing RNA methylation sites or modifications induced secondary structural changes (12). The core reader family includes YTH domain proteins (YTHDF1-3/YTHDC1-2) that directly bind to m6A to regulate mRNA metabolic fate, HNRNPs (Heterogeneous nuclear ribonucleoproteins) family that mediates alternative splicing through structure sensing, and IGF2BPs subfamily that enhances transcript stability (12). The functional activity of these readers is precisely regulated by ubiquitination, forming a complex carcinogenic network.

The YTHDF family, as a key oncogene, is abnormally overexpressed in various tumors (38), and its ubiquitination regulation exhibits cancer species specificity. YTHDF1 is degraded by FBXW8 (F-box and WD repeat domain containing) mediated K11-linked ubiquitination at multiple sites including K469/K473/K500/K527 sites in hepatocellular carcinoma (HCC) (39), but accumulates and promotes metastasis in renal clear cell (RCC) due to low expression of STUB1 (40); At the same time, STUB1 can also target the K245 site of YTHDF2, inducing its degradation and inhibiting the resistance of HCC cells to sorafenib (41), while FBW7 (F-box and WD repeat domain containing 7) in OC and KRT17 (Keratin 17)-SKP2 (S-phase kinase–associated protein 2) complex in CRC inhibit YTHDF2 anti-cancer function by promoting its ubiquitination and degradation (42, 43). Although a tumor suppressor in bladder cancer (BC), YTHDC1 is targeted for degradation via ubiquitination at K372/K469/K472 by the E3 ligases RNF183 (Ring finger protein 183) and XIAP (X-linked inhibitor of apoptosis) (44–46).

All members of the IGF2BPs family have carcinogenic effects and play an extremely important role in the occurrence and development of tumors (47). Meanwhile, the ubiquitination network of the IGF2BPs family is complex and functionally diverse, with the functional consequence being critically determined by the linkage type of the ubiquitin chain. While K48-linked ubiquitination typically targets proteins for proteasomal degradation, as seen with TRIM29-mediated degradation of IGF2BP1 at K440/K450 in GC (48) and LTN1 (Listerin) -mediated degradation in HCC (49), K63-linked ubiquitination often serves non-proteolytic functions to modulate protein activity and complex assembly. In fatty HCC, the E3 ligase RNF5, upregulated via PPARγ activation under conditions of excess free fatty acids, catalyzes K63-linked ubiquitination of IGF2BP1 (50). This modification enhances IGF2BP1’s m6A reader activity specifically towards *CPT1A* mRNA, stabilizing this transcript encoding the rate-limiting enzyme in fatty acid β-oxidation and thereby promoting metabolic adaptation in fatty HCC (50). Similarly, FBXO45, frequently elevated in conventional HCC, catalyzes K63-linked ubiquitination of IGF2BP1 at K190 and K450, leading to IGF2BP1 activation and subsequent stabilization of *PLK1 (Polo-like kinase 1)* mRNA, thereby driving cell proliferation and liver tumorigenesis (51). These parallel mechanisms reveal a recurring theme where specific E3 ligases exploit K63 chains to potentiate the oncogenic function of IGF2BP1 in a context-specific manner. The regulatory landscape of other IGF2BP family members demonstrates similar complexity.

IGF2BP2 undergoes context-specific regulation by different E3 ligases. While IGF2BP2 is targeted for degradation by TRIM21 in CRC and FBW7 in lung cancer (LC), TRIM15 promotes tumor progression through a unique activation mechanism in PC (19, 52, 53). TRIM15 catalyzes K63-linked ubiquitination of IGF2BP2, which induces liquid-liquid phase separation and enhances IGF2BP2’s RNA-binding activity (52). The activated IGF2BP2 subsequently stabilizes *TLR4* mRNA through m6A recognition, establishing a novel TRIM15-IGF2BP2-TLR4 axis that drives pancreatic cancer pathogenesis and chemoresistance (52).Targeted degradation of IGF2BP3 by tissue-specific E3 ligases, Parkin in CC(cervical cancer), TRIM25 in GC, HECTD4 in gliomas, mediates anticancer effects.(54–56).

Meanwhile, non-coding RNA plays a crucial role in cancer by finely regulating the ubiquitination process of the IGF2BPs family. Among them, various circular RNAs and long non-coding RNAs stabilize IGF2BPs by inhibiting the ubiquitination pathway: Hsa_circ_0058495 stabilizes IGF2BP2 by protecting it from TRIM25-mediated ubiquitination and degradation, thereby amplifying downstream oncogenic signaling in PC (57); circEZH2 promotes malignant phenotype by inhibiting the ubiquitination degradation of IGF2BP2 (58); LncRNA LINRIS drives glycolysis reprogramming by stabilizing IGF2BP2 and inhibiting its ubiquitination (59); CircRHBDD1 inhibits its ubiquitination by directly binding to IGF2BP2, thereby promoting tumor progression (60); CircNFATC3 maintains the stability of IGF2BP3 by blocking the activity of E3 ligase TRIM25 (55); CircNEIL3 stabilizes IGF2BP3 by antagonizing the degradation pathway mediated by HECTD4 (HECT domain E3 ubiquitin protein ligase 4) (56). On the contrary, another type of non-coding RNA promotes the ubiquitination degradation of IGF2BPs: lncRNA ABHD11-AS1 accelerates its ubiquitination degradation by promoting the binding of TRIM21 to IGF2BP2 (19); CircNDUFB2, as a scaffold protein, enhances TRIM25 mediated ubiquitination degradation of IGF2BPs and exerts anticancer effects in non-small cell LC (61); LINC01133 directly interacts with IGF2BP2 protein to promote its ubiquitination degradation, thereby inhibiting the progression of estrogen receptor positive BCa (62).

HuR/ELAVL1 (Human antigen R/Embryonic lethal abnormal vision-like 1), as a procancer protein, is ubiquitinated by β-TrCP1 (β-transducin repeat containing E3 ubiquitin protein ligase 1) in multiple tumor tissues (63–65). In CRC and PCa, circRHOBTB3/lncRNA OCC-1 enhances this β-TrCP1-mediated ubiquitination (64, 65). However, circINADL/circUSP1 antagonizes this process in NPC and GC (66, 67). ARPC1B suppresses TRIM21-dependent ubiquitination of HuR (K182 site) in glioblastoma, whereas Pirh2 exerts anticancer effects through its own ubiquitin-mediated degradation pathway(68, 69). The lncRNA ASB16-AS1 inhibits β-TrCP1 to maintain HuR stability and promote the progression of adrenal cortical cancer, while AFAP1-AS1 enhances the ubiquitination degradation of HuR mediated by SMURF1 and accelerates pituitary tumor metastasis (70, 71).

The hnRNP family drives tumor progression by regulating splicing reprogramming, and its ubiquitination modification has subtype specificity (72). ZFP91 (Zinc finger protein 91) mediates the ubiquitination and degradation of hnRNP F at the K185 site in GC and hnRNP A1 at the K8 site in HCC to inhibit tumor metastasis (73, 74). Beyond classical degradation signals, SPSB1 (SPRY domain-containing SOCS box protein 1) catalyzes K29-linked polyubiquitination of hnRNP A1 at K183/K298 sites, inducing its nuclear export and promoting alternative splicing of target mRNAs to accelerate tumor progression (75). FBXO11 (F-box protein 11) promotes the ubiquitination degradation of HnRNP A2B1 at the K27/K48 site, thereby inhibiting the proliferation of HCC cells (76). VHLα promotes the degradation of HnRNP A2B1 at the K274/K305 site, thus inhibiting the proliferation of RCC (77). The anti-cancer function of hnRNP E1 in BCa is reversed by ARIH1 (Ariadne RBR E3 ubiquitin protein ligase 1)-mediated ubiquitination and degradation, while the oncogenic effect of hnRNP L in OC is inhibited by FBXO16-meidated ubiquitination and degradation (78, 79).

### 2.2 The ubiquitination of RMPs in other RNA modifications

Beyond m6A, multiple RNA modifications—including m5C, ac4C, and A-to-I editing—are dynamically regulated by ubiquitination networks in cancer pathogenesis (**Table 2**) (10). The m5C modification, characterized by methylation at the fifth cytosine residues, is frequently elevated in malignancies and orchestrated by a conserved “writer-eraser-reader” system analogous to m6A (8, 80). However, compared to m6A, the ubiquitination regulation of m5C-related proteins remains less extensively characterized, with current evidence primarily focusing on writers and readers.

**Table 2:** Ubiquitin-mediated regulation of RMPs involved in other RNA modifications.

RMP category	RMP	E3 ligase/regulator	Ubiquitination site	Cancer type	Effect on RMP	Functional outcome	Reference
m5C Writer	NSUN2	lincRNA DIAPH2-AS1	K577/K579	GC	Stabilization (inhibits Ub)	Promotes progression via m5C-NTN1 axis	(81)
		LINC00618	–	HCC	Stabilization (inhibits Ub)	Enhances cholesterol biosynthesis via m5C-SREBP2	(82)
		STUB1	K511	BC	Stabilization	Promotes nuclear translocation and subsequent m5C modification of target transcripts in BC	(83)
	NSUN6	MARCH8	K271/K462	Osteosarcoma	Degradation	Promotes the m5C modification of PEX1 and PEX3 mRNA, thereby downregulating intracellular ROS and enhancing the sensitivity of osteosarcoma cells to cisplatin	(84)
m5C Reader	YBX1	SIAH1	K304	OC	Degradation	Enhances cisplatin sensitivity	(85)
		PRP19, TRIM29		HCC	Degradation	Overcomes targeted therapy resistance	(86, 87)
		SMURF2	–	BCa	Degradation	Suppresses taxol resistance	(89)
		Nedd4L	–	CRC, PC	Degradation	Impedes cancer progression	(88, 90)
		TRIM31	K81	CRC	Stabilization	Promotes the proliferation and metastasis of CRC	(91)
		circPRKCA	–	ESCC	Stabilization	Enhances the stability of CSF2 mRNA and promotes the metastasis of ESCC	(92)
ac4C Writer	NAT10	ZSWIM6		HNSCC	Degradation	Inhibits metastasis (pathway suppressed by RNPS1)	(93)
		UBR5	K426	CRC	Degradation	Potentiates EGFR-TKI efficacy	(94)
A-to-I Eraser	ADAR1 (p110)	SMURF2	K744	BCa, CRC	Oligoubiquitination at (stabilization)	Stabilizes oncoprotein, drives transcriptome reprogramming	(97)

#### 2.2.1 The ubiquitination of writers in m5C

As the predominant m5C writer, NSUN2 undergoes cancer type-specific ubiquitination regulation. In GC, lincRNA DIAPH2-AS1 stabilizes NSUN2 by masking its ubiquitination sites (K577/K579), thereby promoting tumor progression through m5C-mediated stabilization of *NTN1* mRNA (81). In HCC, LINC00618 enhances cholesterol biosynthesis by inhibiting ubiquitination of NSUN2 and augmenting m5C modification of *SREBP2* mRNA (82). Beyond stability regulation, KIF26B recruits the STUB1 to catalyze K63-linked ubiquitination of NSUN2 at K511 site, promoting its nuclear translocation and subsequent m5C modification of target transcripts in BC (83). This NSUN2/m5C axis upregulates IL-6 expression, with secreted IL-6 activating STAT3 signaling to enhance transcription of KIF26B while also recruiting DLAT to acetylate NSUN2 and strengthen its interaction with KIF26B (83). This positive feedback loop establishes a self-perpetuating circuit that promotes BC progression and highlights NSUN2 as a potential therapeutic target.

Another m5C writer, NSUN6, is regulated by the E3 ligase MARCH8 (membrane-associated RING-CH-type finger 8) in osteosarcoma (84). MARCH8 ubiquitinates NSUN6 at K271 and K462, leading to its proteasomal degradation (84). Reduced NSUN6 expression decreases m5C modification on PEX1 and PEX3 mRNAs, which downregulates peroxisome synthesis and catalase production (84). The resulting ROS accumulation and DNA damage enhance cisplatin sensitivity, demonstrating a functional interplay between ubiquitination, m5C modification, and chemoresistance.

#### 2.2.2 The ubiquitination of readers in m5C

The primary m5C reader protein YBX1’s therapeutic response is critically regulated by ubiquitination. In OC, SIAH1 (Seven in absentia homolog 1)-mediated ubiquitination at K304 promotes YBX1 degradation, thereby enhancing cisplatin sensitivity (85). Conversely, in HCC, PRP19 (Pre-mRNA processing factor 19) and TRIM29 modulate YBX1 ubiquitination to overcome targeted therapy resistance (86, 87). Additionally, SMURF2 (SMAD specific E3 ubiquitin protein ligase 2) and Nedd4L (Neural precursor cell expressed developmentally down-regulated 4-like) suppress taxol resistance in BCa and impede progression in CRC and PC by mediating the degradation of YBX1 (88–90). Beyond classical degradative ubiquitination, TRIM31 interacts with YBX1 and catalyzes K63-linked polyubiquitination at K81 site, leading to YBX1 protein stabilization. Stabilized YBX1 subsequently enhances the stability of mRNAs encoding EREG, GAS6, and MAFG through both m5C site-dependent and -independent recognition mechanisms, thereby promoting CRC cell proliferation and metastasis (91). Furthermore, non-coding RNAs add another layer of regulation: in ESCC, circPRKCA binds to YBX1 in the cytoplasm, preventing its ubiquitination-mediated degradation. Stabilized YBX1 then increases the stability of CSF2 mRNA in an m5C-dependent manner, thereby facilitating ESCC metastasis (92). These examples illustrate that the ubiquitination of YBX1, whether leading to degradation or functional activation, profoundly impacts m5C-dependent gene expression and tumor progression.

#### 2.2.3 The ubiquitination of other RMPs

The acetylated modification ac4C, the only known RNA acetylation in eukaryotes, plays pivotal roles in tumorigenesis through its exclusive writer NAT10 (3, 8). In head and neck squamous cell carcinoma (HNSCC), RNPS1 (RNA-binding protein with serine-rich domain 1) competitively inhibits ZSWIM6 (Zinc finger SWIM-type containing 6)-mediated ubiquitination of NAT10, leading to aberrant tRNA ac4C modification and metastatic progression (93). Contrastingly, in CRC, 5-Fluorouracil enhances UBR5 (Ubiquitin Protein Ligase E3 Component N-Recognin 5)-dependent ubiquitination of NAT10 at K426, suppressing ac4C modification of *ERRFI1* mRNA and potentiating EGFR-TKI efficacy through EGFR pathway activation (94).

A-to-I RNA editing, catalyzed by adenosine deaminases acting on RNA (ADARs), exhibits extensive dysregulation in tumors (3, 8). The constitutively expressed ADAR1 isoform p110 acts as an oncoprotein in both BCa and CRC.(95, 96). Notably, SMURF2 stabilizes ADAR1p110 by mediating oligoubiquitination at K744, which impedes both proteasomal and lysosomal degradation pathways (97). This ubiquitin-dependent stabilization mechanism exemplifies cross-regulation between ubiquitination and RNA editing machinery, driving cancer-specific transcriptome reprogramming.

### 2.3 Epitranscriptomic regulation of ubiquitin ligase mRNAs

The RNA modification apparatus establishes a robust feedback loop that modulates the ubiquitin system by controlling the stability and translation of E3 ligase mRNAs, thereby forming a tightly interconnected regulatory network (**Figure 2** and **Table 3**). This reciprocal crosstalk is orchestrated through epitranscriptomic pathways—predominantly m6A—wherein reader proteins act as key interpreters that determine the fate of ubiquitin ligase transcripts. The epitranscriptomic fate of an E3 ligase mRNA is largely dictated by which reader protein recognizes its m6A marks. This creates a regulatory logic where the same modification can lead to opposite outcomes depending on the reader context.

**Table 3:** Epitranscriptomic regulation of E3 ubiquitin ligase mRNAs.

Category	E3 ubiquitin ligase	Regulating RMP(s)	Cancer type	Type of regulation	Functional outcome	Reference
m6A Reader-Mediated						
IGF2BP-Stabilized	MIB1	METTL3/IGF2BP2	GC	mRNA stabilization	Potentiate peritoneal metastasis	(98)
	MIB1	IGF2BP3	Glioma	mRNA stabilization	Promote tumor progression	(36)
	FBXO43	METTL3/IGF2BP2	HCC	mRNA stabilization	Promote proliferation and invasion	(99)
	WWP2	METTL3/IGF2BP2	HCC	mRNA stabilization	Confer doxorubicin resistance	(100)
	TRIM11	METTL3/IGF2BP1	NSCLC	mRNA stabilization	Promote malignant progression	(101)
	TRIM47	METTL3	OC	mRNA stabilization	Contribute to paclitaxel resistance	(102)
	TRIM11	METTL14/IGF2BP1	CC	mRNA stabilization	Promote tumor growth	(103)
	ZFP14	METTL14/IGF2BP2	RCC	mRNA stabilization	Enhance oncogenic expression	(138)
	Nedd4	METTL14	HCC	mRNA stabilization	Promote TGF-β signaling and tumor progression	(139)
YTHDF2-Degraded	TRIM21	YTHDF2	HCC	mRNA decay	Accelerate cancer progression	(20)
	TRIM50	YTHDF2	GC	mRNA decay	Remove tumor suppressive restraint	(105)
	FBXW7	YTHDF2	LUAD	mRNA decay	Promote tumor growth	(106)
	TRIM7	YTHDF2	Osteosarcoma	mRNA decay	Inhibit tumor development	(107)
	Siah2	METTL14/YTHDF2	CCA	mRNA decay	Regulate tumor progression	(108)
	TRIM13	RBM15/YTHDF2	NSCLC	mRNA decay	Promote cancer progression	(109)
YTHDF1-Stabilized	FBXO31	METTL3-METTL14/YTHDF1	PC	Translation enhancement	Promote tumor growth	(110)
	UBE3C	METTL5/YTHDF1	Osteosarcoma	mRNA protection	Promote tumor growth	(111)
	TRIM16	METTL14/YTHDF1	Triple-negative BCa	mRNA stabilization	Trigger FGF7 ubiquitination and degradation	(112)
YTHDF3-Regulated	SPOP	YTHDF3	PCa	mRNA decay	Facilitate cancer progression	(113)
	TRIM8	YTHDF3	PCa	mRNA stabilization	Modulate degradation pathways	(114)
Eraser-Modulated						
FTO-Targets	FOXO3a	FTO	PCa	mRNA stabilization (via demethylation)	Promote apoptosis and suppresses proliferation	(35)
ALKBH5-Targets	UBR7	ALKBH5	HCC	mRNA stabilization	Inhibit proliferation	(115)
	RNF40	ALKBH5	Osteosarcoma	mRNA stabilization	Promote tumorigenesis	(116)
	TRIM8	ALKBH5	PCa	Translation suppression	Regulate oncogenic signaling	(114)
Other Modifications						
Ψ-Mediated	MIB1	DKC1/SNORA73B	Various Cancers	mRNA stabilization	Promote tumor progression	(117)
ac4C-Mediated	Mdm2	NAT10	GC	mRNA stabilization	Accelerate p53 degradation	(118)
m5C-Mediated	TRIM28	NSUN2/YBX1	PCa	mRNA stabilization	Promote proliferation and metastasis	(121)
	RNF115	YBX1	HCC	mRNA stabilization	Promote proliferation and metastasis	(122)

#### 2.3.1 The IGF2BP family: A master switch for mRNA stabilization and oncogenic reinforcement

The IGF2BP family (IGF2BP1/2/3) consistently functions as a stabilization module upon recognizing m6A modifications. By binding to m6A sites, typically in the 3’UTR, these readers protect target transcripts from degradation, thereby enhancing the expression of the encoded proteins. This mechanism is frequently hijacked in cancer to reinforce oncogenic signaling by stabilizing the mRNAs of pro-tumorigenic E3 ubiquitin ligases. For instance, in GC, HCC, NSCLC, and OC, the METTL3-IGF2BP axis stabilizes mRNAs of oncogenic E3 ligases like MIB1, FBXO43, WWP2, TRIM11, and TRIM47 (98–102). This principle extends to other writers, as METTL14-IGF2BP-mediated stabilization of ZFP14 and TRIM11 mRNA promotes RCC and CC progression (103). Thus, the IGF2BP family serves as a critical node that amplifies oncogenic ubiquitin ligase activity through post-transcriptional mRNA stabilization.

#### 2.3.2 The YTHDF family: A tripartite system for mRNA decay, translation, and context-dependent regulation

In contrast to the stabilizing function of IGF2BPs, the YTHDF family members regulate ubiquitin ligase levels in a specialized and context-dependent manner.

YTHDF2 predominantly mediates mRNA decay by recognizing m6A-modified transcripts and orchestrating their degradation through recruitment of the CCR4-NOT deadenylate complex (104). This degradation function predominantly affects tumor-suppressive E3 ligases, including *TRIM21* in HCC, where its decay accelerates cancer progression by reducing TRIM21-mediated degradation of oncoproteins (20). Similarly, YTHDF2 mediates decay of *TRIM50* mRNA in GC and *FBXW7* mRNA in lung adenocarcinoma (LUAD), removing critical restraints on tumor growth (105, 106). In osteosarcoma, YTHDF2-mediated decay of *TRIM7* mRNA contributes to inhibition of tumor development, demonstrating that its targets include both oncogenic and tumor-suppressive factors depending on cellular context (107). METTL14/YTHDF2-controlled *Siah2* mRNA decay in CCA (108). Additional complexity arises from cross-regulation within the m6A machinery, as exemplified by RBM15-mediated m6A modification downregulating *TRIM13* mRNA expression in a YTHDF2-dependent manner, thereby promoting NSCLC progression (109).

YTHDF1 generally exerts stabilizing and translation-enhancing effects through its interaction with the translation initiation machinery, playing a critical role in maintaining the expression of E3 ligases involved in cell cycle regulation and the DNA damage response. This function is demonstrated across multiple cancer types: in PC, METTL3/METTL14-mediated m6A modification promotes *FBXO31* mRNA translation in a YTHDF1-dependent manner (110); in osteosarcoma, METTL5-mediated m6A modification enables YTHDF1 binding and protection of *UBE3C* mRNA from degradation (111); and in triple-negative BCa, hypoxia-induced exosomal METTL14 enhances *TRIM16* mRNA stability through YTHDF1, leading to TRIM16-mediated ubiquitination and degradation of FGF7 (112). This stabilization function appears to be particularly important for maintaining the expression of E3 ligases involved in cell cycle regulation and DNA damage response.

YTHDF3 demonstrates the most complex regulatory pattern within the YTHDF family, exhibiting context-dependent dual functions that can either promote or suppress tumor growth. In PCa, YTHDF3 drives *SPOP* mRNA decay, reducing the abundance of this critical tumor suppressor and facilitating cancer progression (113). Paradoxically, within the same cancer type, YTHDF3 can also stabilize *TRIM8* transcripts through m6A recognition, highlighting its capacity for both oncogenic and tumor-suppressive activities depending on specific cellular contexts and co-factor interactions (114). This functional duality suggests that YTHDF3 may serve as a regulatory switch that can modulate the balance between different ubiquitin-mediated degradation pathways.

#### 2.3.3 m6A erasers (FTO and ALKBH5): sculpting the reader landscape

The m6A erasers, FTO and ALKBH5, add another layer of regulatory logic by dynamically sculpting the epitranscriptomic landscape. They do not directly bind the mRNA fate, but rather function as gatekeepers that determine whether a transcript becomes a substrate for the reader modules described above. By removing m6A marks, they can either expose transcripts to decay or protect them from it, depending on the dominant reader in that context. For example, in PCa, FTO-mediated demethylation of *FOXO3a* mRNA prevents its recognition by the decay-promoting reader YTHDF2, leading to its stabilization and a tumor-suppressive outcome (26). Conversely, in osteosarcoma, ALKBH5 removes m6A from *RNF40* mRNA, reducing its YTHDF3-mediated degradation and thereby promoting tumorigenesis (107). This positions erasers not merely as passive removers of marks, but as active regulators that can toggle the switch between different m6A reader outcomes.

#### 2.3.3 m6A erasers (FTO and ALKBH5): sculpting the reader landscape

The regulatory landscape extends beyond simple binary outcomes, exhibiting sophisticated context-specificity and even paradoxical behaviors that are further complicated by the involvement of m6A erasers such as ALKBH5 and FTO. As key m6A demethylases, they dynamically sculpt the epitranscriptome by removing methyl groups from adenosine, thereby fundamentally altering the recognition landscape for reader proteins and consequently changing the fate of key regulatory mRNAs. This eraser function demonstrates remarkable cancer type-specific duality.

In PCa, FTO-mediated demethylation stabilizes the mRNA of the tumor suppressor transcription factor *FOXO3a (*35). By erasing m6A marks on *FOXO3a* transcripts, FTO prevents their recognition and degradation by decay-promoting readers (YTHDF2), leading to enhanced FOXO3a protein expression (35). Conversely, in HCC, *ALKBH5* deficiency elevates m6A modification levels on *UBR7* mRNA, enhancing its recognition by stabilizing readers and consequently stabilizing this tumor suppressor transcript to inhibit proliferation (115). And, in osteosarcoma, ALKBH5 overexpression reduces m6A methylation of *RNF40* mRNA, diminishing YTHDF3-mediated degradation and thereby promoting tumorigenesis through increased RNF40 abundance (116). The paradoxical relationship extends to PCa, where ALKBH5 downregulation enhances m6A methylation of *TRIM8* mRNA to suppress its translation, while in the same biological context, YTHDF3 can stabilize *TRIM8* transcripts through m6A recognition, creating a complex regulatory interplay between erasers and readers (114). These findings position ALKBH5 and FTO not merely as a passive eraser but as an active regulator that determines the specificity and outcome of m6A-mediated regulation of the ubiquitin system, with its activity ultimately shaping the oncogenic trajectory through precise control of E3 ligase expression.

#### 2.3.4 Integration with other RNA modifications (Ψ, ac4C, m5C)

While m6A provides the most extensively characterized regulatory logic, other modifications integrate into this network through distinct mechanisms. The Ψ synthase DKC1, guided by snoRNAs like SNORA73B, enhances the stability of target mRNAs such as the oncogenic E3 ligase *MIB1* (108). The ac4C writer NAT10 can enhance mRNA stability, as seen with *Mdm2* in GC (109), adding another layer of complexity. Furthermore, the m5C writer NSUN2 and its reader YBX1 form a functional pair analogous to the m6A writer-reader systems. This pair stabilizes mRNAs of E3 ligases like *TRIM28* in PCa and *RNF115* in HCC, demonstrating that the principle of “writer-installs, reader-interprets, and dictates mRNA fate” is a recurring theme across multiple epitranscriptomic marks (112).

#### 2.3.4 Regulation by other RNA modifications (Ψ, ac4C, m5C)

Beyond m6A, Ψ, ac4C and m5c critically regulate RNA biogenesis, structure, and stability (8). The Ψ synthase DKC1 catalyzes Ψ deposition to enhance mRNA stability and translation efficiency (8). Specifically, SNORA73B guides site-specific Ψ modification of *MIB1* mRNA through base-pairing interactions with DKC1, further stabilizing *MIB1* transcripts to increase protein abundance and promote tumor progression (117). In *Helicobacter pylori*-associated GC, bacterial infection induces NAT10 expression, which promotes ac4C modification of *Mdm2* mRNA to stabilize transcripts (118), thus leading to upregulated Mdm2 protein expression and accelerated p53 degradation, thereby modulating GC progression (118). Interestingly, NAT10 paradoxically functions as an E3 ubiquitin ligase that mediates the ubiquitination and degradation of Mdm2 (119). TRIM28 is a protein with ubiquitinated E3 enzyme activity and plays a pro-cancer role in PCa (120). Wang et al. found that NSUN2-mediated m5C methylation of *TRIM28* mRNA is recognized by YBX1, which in turn stabilizes the transcript. This mechanism consequently enhances the proliferation and metastasis of PCa (121). Another paradigm emerges from the interplay among m5C modification, YBX1, and the E3 ligase RNF115 in HCC. NSUN2-mediated m5C modification of RNF115 mRNA enhances its translation in a YBX1-dependent manner (122). YBX1 binds to m5C-modified sites within the *RNF115* 3’-UTR, promoting mRNA circularization and translational efficiency (122). The resultant upregulation of RNF115 targets dihydroorotate dehydrogenase (DHODH) for K27-linked ubiquitination, inhibiting its autophagic degradation and suppressing ferroptosis to promote HCC progression (122).

## 3 Therapeutic targeting of the ubiquitination-epitranscriptome axis

**Figure 3:**
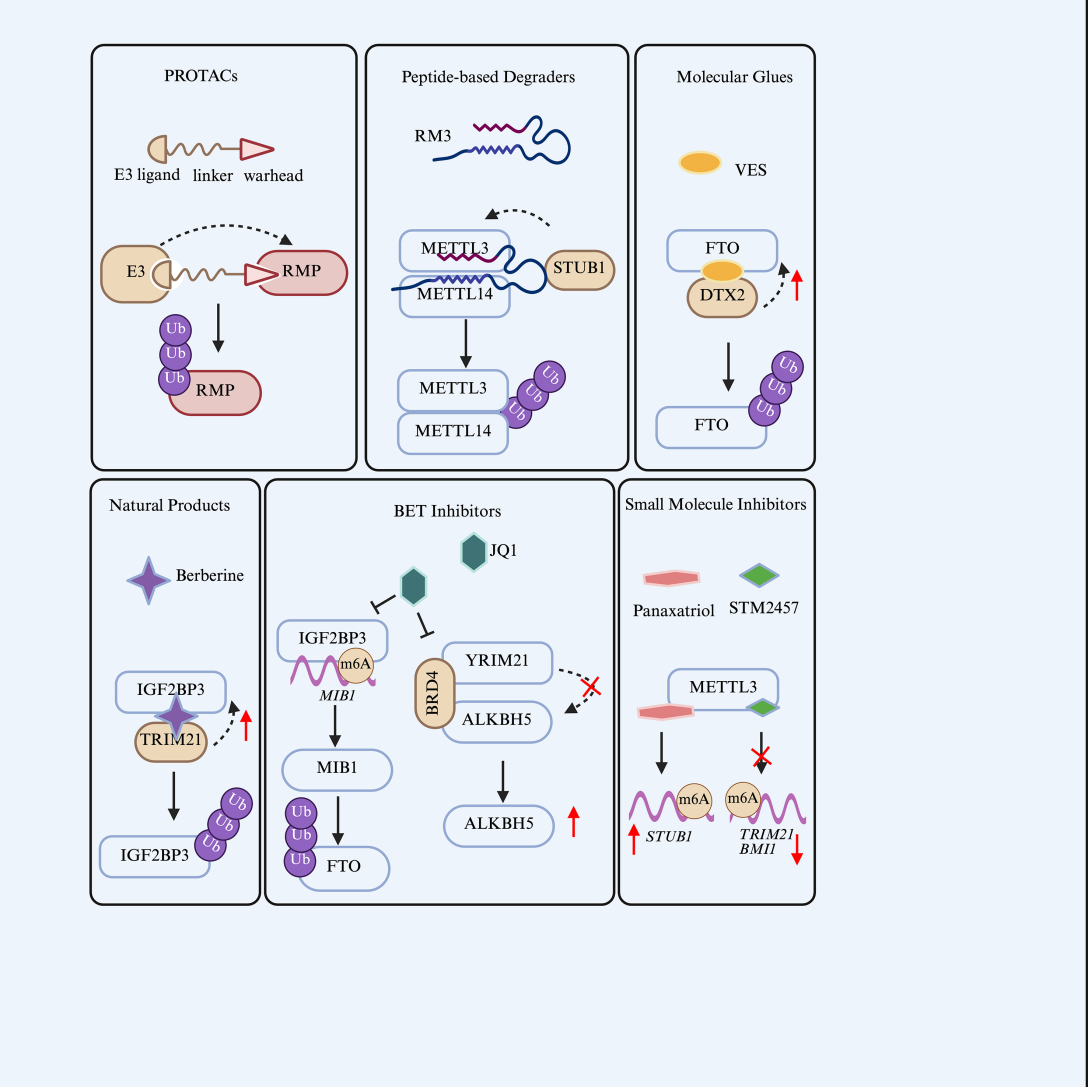
Therapeutic strategies targeting the ubiquitination-epitranscriptome axis.

The dynamic crosstalk between ubiquitination and RNA modifications generates unique therapeutic vulnerabilities in cancer. Targeting this axis presents innovative opportunities to disrupt oncogenic signaling, overcome therapy resistance, and restore antitumor immunity. Therapeutic strategies can be broadly divided into two complementary approaches: 1) direct degradation of RMPs via the ubiquitin system, and 2) modulation of RMP activity to indirectly influence the ubiquitin ligase network (**Figure 3**). While these strategies have demonstrated preclinical promise, critical evaluation of their translational potential, specificity, and limitations is essential for guiding future therapeutic development.

### 3.1 Direct targeting: ubiquitin-mediated degradation of RMPs

This approach leverages the ubiquitin-proteasome system to directly degrade oncogenic RMPs, offering potential advantages over conventional inhibition strategies—particularly for targets with scaffolding functions or poorly druggable active sites. However, the clinical translation of these degraders faces substantial challenges, including optimal bioavailability, off-tissue toxicity, and the emergence of resistance mechanisms.

#### 3.1.1 PROTACs for m6A regulator degradation

Proteolysis-targeting chimeras (PROTACs) constitute a groundbreaking degradation technology that enables precise reprogramming of ubiquitination-RNA modification networks through rationally designed bifunctional molecules. Each PROTAC consists of three key elements: a ligand that binds the target protein, a recruiter for an E3 ubiquitin ligase, and a linker that optimizes spatial conformation (123). By simultaneously engaging both the target proteins and E3 ligases, PROTACs catalyze K48-linked polyubiquitination, directing substrates to 26S proteasomal degradation (124). This event-driven mechanism enables single PROTAC molecules to undergo multiple catalytic cycles, overcoming the occupancy-driven limitations of traditional inhibitors—particularly effective against non-enzymatic scaffolds like METTL14 or multi-subunit complexes (125). Several PROTACs have been developed to specifically degrade m6A-related RMPs, holding significant promise for cancer therapy (**Table 4**).

**Table 4:** PROTACs targeting m6A RMPs.

Compound	Target	Warheads	E3 ligase	E3 ligand	IC50/EC50	DC50	Cell/model	Ref
ZW27941	METTL3	UZH2	VHL	4W9H	MV4.11: IC50 = 1.65μM; MOLM13: IC50 = 3.31μM; NB4: IC50 = 9.1μM	MV4.11: DC50 = 0.70μM; MOLM13: DC50 = 0.17μM; NB4: DC50 = 0.13μM	*In vitro*: MOLM13, MV4.11, NB4	(129)
WD6305	METTL3/METTL14	UZH2	VHL	(S, R, S)-AHPC-Me	MOLM-13: IC50 = 0.78μM	Mono-Mac-6: DC50 = 0.14 μM(METTL3); DC50 = 0.194μM (METTL14)	*In vitro*: Mono-Mac-6, MOLM-16	(125)
KH12	METTL3	UZH2	VHL	VH032	MOLM-13: IC50 = 1.04μM; AGS: IC50 = 1.48μM; SNU484: IC50 = 3.00μM	MOLM-13: DC50 = 0.22 μM	*In vitro*: MOLM-13, AGS, SNU484; Organoids derived from gastric cancer patients	(128)
AF151	METTL3	STM2457	VHL	VH032	MOLM-13: IC50 = 0.45 μM	MOLM-13: DC50 = 0.43 μM	*In vitro*: MOLM-13	(140)
PROTACs 22, 24, 30	METTL3	UZH2	CRBN	Pomalidomide	MOLM-13: IC50 = 0.19μM(22); IC50 = 0.47μM (24); IC50 = 0.07μM(30)	–	*In vitro*: PC3	(127)
QP73	FTO	FB23	CRBN	Thalidomide	NB4: IC50 = 0.026μM; NOLM13: IC50 = 0.108μM; HEL: IC50 = 0.102μM; NOMO1: IC50 = 0.027μM; KG-1: IC50 = 0.013μM; MV4-11: IC50 = 0.026μM	NB4: DC50 = 0.0349 μM; MV4-11: DC50 = 65.9 nmol/L	*In vitro*: NB4, HEL, NOMO1, KG-1, MV4-11; NB4 and MV4–11 xenograft mouse model	(130)
PROTACs 1a and 1c	YTHDF2	4RDN	CRBN	Pomalidomide	–	–	*In vitro*: K562	(132)
bioPROTAC	HuR	VHH	TRIM21	T21RBCC	–	–	*In vitro*: HCT116; HCT116 xenograft mouse model	(133)
VES (Molecular Glue)	FTO		DTX2		CHL1 cells IC50 = 12.3μM		*In vitro*: CHL1	(34)

UZH2, a selective METTL3 inhibitor, has been employed as a ligand module in multiple PROTAC designs (126). Errani et al. developed a series of METTL3 degraders by conjugating the CRBN ligand pomalidomide with UZH2 via systematically optimized linkers (127). While derivatives 22, 24, and 30 showed enhanced anti-proliferative efficacy in prostate cancer PC3 cells compared to other analogs, the tissue-specific response heterogeneity observed raises important questions about the determinants of PROTAC activity across different cellular contexts (128). Similarly, Du et al. designed WD6305, which induces dose-dependent METTL3 degradation in acute myeloid leukemia cells and concurrently depletes METTL14, confirming disruption of the METTL3-METTL14 complex stability (125). However, whether such co-degradation represents a therapeutic advantage or a source of unintended off-target effects remains to be determined, given the context-dependent tumor-suppressive functions of METTL14 in certain malignancies.

The KH12 PROTAC developed by Hwang et al. incorporates a heptane linker to enhance VHL recruitment efficiency and demonstrates efficacy in both AML xenografts and gastric cancer patient-derived organoids (128). While this broader applicability is encouraging, the differential sensitivity between AML and solid tumor models suggests that tumor-intrinsic factors—including baseline E3 ligase expression, proteasome capacity, and membrane permeability—may profoundly influence therapeutic response. Mechanistically, ZW27941, a VHL-engaging degrader developed by Nar et al., induces co-degradation of METTL3/METTL14 and acts synergistically with standard therapies, providing a strategy to overcome treatment resistance (129). Nevertheless, the therapeutic window and potential for on-target toxicity in normal tissues expressing METTL3 have not been systematically evaluated.

For RNA demethylation erasers, the FTO-targeting PROTAC QP73 demonstrates 100-1000-fold greater anti-proliferative activity than the conventional inhibitor Dac85 in AML cells, with significant tumor growth suppression in xenograft models (130, 131). This dramatic efficacy enhancement exemplifies the catalytic advantage of PROTACs over occupancy-driven inhibitors. However, the extent to which QP73 selectively targets FTO, and whether FTO degradation in non-malignant tissues produces unintended consequences, requires rigorous investigation. Targeting m6A readers, Goebel et al. developed YTHDF2-degrading PROTACs 1a and 1c that effectively disrupt m6A RNA-binding function in chronic myeloid leukemia K562 cells (132). While RNA immunoprecipitation assays confirmed target engagement, the functional consequences of YTHDF2 loss in normal hematopoiesis—where it plays essential physiological roles—remain unexplored, highlighting a critical gap in assessing therapeutic index.

Complementing small-molecule approaches, Fletcher et al. developed a TRIM21-based bioPROTAC incorporating an anti-HuR single-domain antibody (VHH) that achieves endogenous HuR degradation, reverses tumor-promoting properties in CRC *in vivo*, and restores chemosensitivity (133). This biologic approach offers superior target specificity but faces challenges related to delivery, immunogenicity, and intracellular accessibility that may limit clinical translation compared to small-molecule counterparts.

#### 3.1.2 Peptide-based degraders and molecular glues

Beyond the bifunctional PROTAC molecules, other innovative modalities are emerging to induce the degradation of RMPs, primarily including peptide-based degraders and molecular glues, which exploit endogenous ubiquitination pathways with unique advantages. Peptide-based strategies present a complementary avenue for therapeutic intervention by mimicking protein interfaces or inducing conformational changes. For instance, the peptide inhibitor RM3 disrupts the METTL3/14 protein-protein interface and promotes STUB1-mediated ubiquitination and degradation of the complex, demonstrating significant anti-tumor activity in melanoma models and synergistic efficacy with anti-PD-1 therapy (134). While conceptually elegant, peptide therapeutics generally suffer from poor pharmacokinetic properties, limited oral bioavailability, and rapid proteolytic degradation—obstacles that must be overcome before clinical application.

Molecular glues represent a distinct class of degraders that function by inducing or stabilizing novel protein-protein interactions between an E3 ligase and a target protein. Vitamin E Succinate (VES) exemplifies this approach, functioning as a heterobifunctional molecular glue degrader for FTO (34). The succinate moiety binds FTO while the vitamin E moiety engages DTX2, promoting FTO ubiquitination and proteasomal degradation (34). he identification of VES provides proof-of-concept that RMPs can be targeted via molecular gluing, expanding the therapeutic arsenal beyond bifunctional PROTACs. However, the relatively weak binding affinity typical of molecular glues may necessitate high concentrations for efficacy, raising concerns about off-target effects and the feasibility of achieving therapeutic doses *in vivo*.

### 3.2 Indirect targeting: modulating RMPs to influence ubiquitin ligase networks

This strategy aims to indirectly modulate the expression and function of ubiquitin ligases and key signaling nodes by targeting RMPs, leveraging the epitranscriptomic control of E3 ligase mRNAs and the regulatory influence of specific RMPs. While conceptually attractive due to the potential for pathway-level effects, this approach faces the inherent challenge of pleiotropy—RMPs regulate numerous transcripts, making pathway-selective modulation difficult to achieve.

Inhibition of METTL3 has emerged as a particularly effective means to regulate oncogenic E3 ligases. The METTL3 inhibitor STM2457 demonstrates broad potential in modulating E3 networks by sensitizing HCC to oxaliplatin through disruption of the METTL3-TRIM21-G6PD axis and suppressing HCC growth by targeting the METTL3-BMI1/RNF2 axis (20, 135). However, the extent to which these effects reflect on-target METTL3 inhibition versus off-target activities, and whether chronic METTL3 inhibition produces compensatory changes in the epitranscriptome, warrant longitudinal investigation. Natural products also offer promising therapeutic opportunities. Panax ginseng-derived 20(R)-panaxatriol (PT) enhances METTL3-mediated m6A modification of *STUB1* mRNA, inhibiting autophagy and exerting antitumor effects in triple-negative BCa (136). Nevertheless, the multifunctional nature of natural products complicates mechanistic attribution, and their generally modest potency presents formulation challenges.

The BET inhibitor JQ1 exemplifies a powerful strategy of targeting upstream epigenetic regulators to control RMP stability, though with notable context-dependent effects. In BCa, JQ1-mediated BRD4 inhibition disrupts a scaffolding function that facilitates TRIM21-ALKBH5 interaction, leading to ALKBH5 stabilization and subsequent *ESPL1* mRNA decay (37). Conversely, in glioma, JQ1 downregulates IGF2BP3 expression, disrupting MIB1-mediated FTO ubiquitination and degradation, thereby reversing oncolytic herpes simplex virus therapy resistance (36). These parallel findings—where JQ1 stabilizes ALKBH5 in BCa and FTO in glioma—collectively highlight the profound therapeutic potential of BET inhibitors in reprogramming RMP ubiquitination networks. However, they also underscore a critical caveat: the effects of such upstream modulators are highly context-dependent, and predictive biomarkers will be essential to identify patients most likely to benefit.

Natural products continue to represent a valuable source for modulating the ubiquitination-epitranscriptome axis. The plant-derived alkaloid berberine (BBR) directly binds IGF2BP3 and induces structural alterations that promote TRIM21-mediated K48-linked ubiquitination and proteasomal degradation, disrupting oncogenic transcripts such as CCND1 and inhibiting CRC progression (137). While berberine’s safety profile is well-established, its relatively low potency and multifactorial mechanisms of action complicate dose optimization and may limit therapeutic windows.

### 3.3 Critical assessment and future directions

Despite the considerable promise of targeting the ubiquitination-epitranscriptome axis, several critical considerations warrant attention. First, the cancer type-specific and context-dependent functions of many RMPs—exemplified by YTHDF3’s dual oncogenic and tumor-suppressive activities within the same tissue (113, 114) —necessitate careful patient stratification and raise concerns about unintended tumor-promoting effects following therapeutic intervention. Second, the physiological functions of RMPs in normal tissues remain incompletely characterized, and the therapeutic index for RMP-targeted degraders has not been systematically established. Third, resistance mechanisms to PROTACs and molecular glues—including mutations in target proteins, E3 ligases, or proteasome components—are likely to emerge and require proactive investigation.

Future directions should include the development of degraders with enhanced selectivity through structure-based design and screening approaches, the identification of predictive biomarkers to guide patient selection, and the exploration of combination strategies with conventional therapies, immunotherapies, and other targeted agents. Rigorous evaluation of on-target toxicity in preclinical models and investigation of resistance mechanisms will be essential to inform next-generation therapeutic design. The convergence of ubiquitination and RNA modification biology offers unprecedented opportunities for therapeutic innovation, but realizing this potential will require balanced appreciation of both opportunities and limitations.

## 4 Conclusion

This review has systematically elucidated the sophisticated bidirectional crosstalk between ubiquitination pathways and RNA modifications in cancer pathogenesis, revealing a complex regulatory network that profoundly influences tumorigenesis, metastasis, and therapeutic response. We have comprehensively examined how ubiquitination precisely controls the stability and activity of RMPs, thereby shaping the epitranscriptomic landscape, while simultaneously exploring how RNA modifications feedback to regulate ubiquitin ligase expression through epitranscriptomic reprogramming. This intricate interplay creates a dynamic system that confers remarkable adaptability and therapeutic resistance to cancer cells, presenting both challenges and opportunities for therapeutic intervention.

Looking forward, several key research directions warrant focused investigation to translate these mechanistic insights into clinical applications. Future studies should prioritize expanding our understanding beyond the well-characterized m6A modification to encompass other RNA modifications such as m5C, ac4C, Ψ, and A-to-I editing, systematically mapping their connections with ubiquitination pathways across different cancer types to identify context-specific vulnerabilities. Concurrently, advancing targeted intervention technologies remains crucial, particularly through the development of more selective and potent degraders targeting specific RMPs, optimizing PROTAC design elements including E3 ligase recruiters and linkers, and exploring novel modalities such as molecular glues and peptide-based degraders. Addressing translational challenges related to bioavailability, tissue specificity, and pharmacokinetic properties will be critical to successfully harnessing this complex regulatory axis in oncology.

The clinical translation of these mechanistic insights urgently requires focused efforts on biomarker development and combination strategies. It is essential to identify predictive biomarkers, whether epitranscriptomic signatures or ubiquitin-related gene expression patterns, will be essential for patient stratification and treatment selection. Furthermore, rationally designed combination therapies that integrate inhibitors targeting this axis with conventional chemotherapy, immunotherapy, or targeted agents must be rigorously evaluated in preclinical models and clinical trials to overcome resistance and improve therapeutic efficacy. Addressing the complexity and context-dependency of this regulatory network demands sophisticated approaches, including the application of single-cell multi-omics and spatial transcriptomics to decipher cell-type-specific and tumor-microenvironment-specific dynamics, thereby ensuring that therapeutic interventions are precisely tailored to the appropriate biological context.

In conclusion, the ubiquitination-epitranscriptome interface represents a master regulatory layer in cancer biology that offers unprecedented opportunities for therapeutic intervention. By fostering interdisciplinary collaboration, we can accelerate the translation of these fundamental discoveries into novel therapeutic strategies that ultimately improve outcomes for cancer patients worldwide.
